# "Time sweet time": circadian characterization of galectin-1 null mice

**DOI:** 10.1186/1740-3391-8-4

**Published:** 2010-04-19

**Authors:** Leandro P Casiraghi, Diego O Croci, Francoise Poirier, Gabriel A Rabinovich, Diego A Golombek

**Affiliations:** 1 Departamento de Ciencia y Tecnología, Universidad Nacional de Quilmes/CONICET, Argentina; 2 Laboratorio de Inmunopatología, Instituto de Biología y Medicina Experimental (IByME)/CONICET, Argentina; 3 Jacques Monod Institute, UMR-CNRS7592, Paris Diderot University, 75205 Paris, France

## Abstract

**Background:**

Recent evidence suggests a two-way interaction between the immune and circadian systems. Circadian control of immune factors, as well as the effect of immunological variables on circadian rhythms, might be key elements in both physiological and pathological responses to the environment. Among these relevant factors, galectin-1 is a member of a family of evolutionarily-conserved glycan-binding proteins with both extracellular and intracellular effects, playing important roles in immune cell processes and inflammatory responses. Many of these actions have been studied through the use of mice with a null mutation in the galectin-1 (*Lgals1*) gene. To further analyze the role of endogenous galectin-1 *in vivo*, we aimed to characterize the circadian behavior of galectin-1 null (*Lgals1*^-/-^) mice.

**Methods:**

We analyzed wheel-running activity in light-dark conditions, constant darkness, phase responses to light pulses (LP) at circadian time 15, and reentrainment to 6 hour shifts in light-dark schedule in wild-type (WT) and *Lgals1*^-/- ^mice.

**Results:**

We found significant differences in free-running period, which was longer in mutant than in WT mice (24.02 vs 23.57 h, p < 0.005), phase delays in response to LP (2.92 vs 1.90 circadian h, p < 0.05), and also in *alpha *(14.88 vs. 12.35 circadian h, p < 0.05).

**Conclusions:**

Given the effect of a null mutation on circadian period and entrainment, we indicate that galectin-1 could be involved in the regulation of murine circadian rhythmicity. This is the first study implicating galectin-1 in the mammalian circadian system.

## Background

Circadian systems represent an endogenous mechanism adapted to cycling environmental conditions. In mammals, the central circadian clock is located in the suprachiasmatic nuclei (SCN), guiding circadian-regulated biological variables such as the sleep-wake cycle, hormonal secretions and locomotor activity [1]. Another physiological process that exhibits circadian fluctuations, with obvious implications in disease progression and outcome, is the regulation of immune function.

The link between the circadian and the immune systems has been extensively investigated [2-4]. Circadian rhythms within the immune system were described in several tissues and cellular populations [5]. In humans, the number of lymphocytes and granulocytes peaks during the night, whereas monocytes and neutrophil levels fall during the day [6]. Major humoral immune responses undergo circadian changes, and rhythms in plasmatic levels of pro-inflammatory cytokines, as well as peptide hormones produced and secreted by immune cells, were also reported [3,5-7].

In addition, circadian outputs might affect the central clock through a feedback mechanism that fine-tunes the system. The influence of the immune over the circadian system has been studied in recent years, and there are evidences of the effects of cytokines or chemokines in the central nervous system (CNS) [8-13]. A growing number of recent evidences point to the existence of a bidirectional interaction between the immune and circadian systems which might be essential in the study of disease progression and treatment. Recent work in our laboratory has shown that acute doses of bacterial LPS induce phase changes in mice [4,14], probably mediated through NF-κB activation [15]. Moreover, cytokines activate clock genes in CNS glial cells, which could release factors to synchronize CNS neurons [16].

Galectins, a family of evolutionarily-conserved β-galactoside-binding proteins, can control a variety of biological processes including the regulation of neuroimmunoendocrine networks [17,18]. Galectin-1 (Gal1), a homodimeric protein of this family, can regulate immune cell homeostasis by modulating cytokine synthesis, T cell survival and dendritic cell physiology (reviewed in [19]). Blockade of Gal1 expression within tumor tissue results in heightened T-cell mediated tumor rejection and increased survival of antitumor Th1 cells [20]. Recent evidence from our laboratory provided rational explanations for these selective immunosuppressive effects demonstrating the ability of Gal1 to preferentially regulate the survival of particularly glycosylated T-cell subsets [21] and induce the amplification of tolerogenic signals delivered from dendritic cells to T cells in a paracrine circuit involving IL-27 and IL-10 [22]. Although the major role for Gal1 has been studied in the context of immune regulation, tumor progression or inflammation [18,22-25], recent evidence suggests that it might also play an important role in nerve regeneration [26], muscle innervation [27] and neurogenesis [28,29]. In fact, Gal1 is expressed in both motor and sensory neurons [30] and modifies the expression of neuronal NMDA glutamate receptors [31]. Galectins have been associated with several biological processes in physiological as well as in pathological conditions (reviewed in [18]); yet the direct involvement of galectins in animal behavior is largely unexplored.

Based on the evidence that several cytokines and immune factors are known to affect circadian rhythms[13,14,32-34], we wished to explore whether the individual functions of Gal1 displayed on different biological settings, may influence or compromise behavioral parameters. The aim of this study is to establish a role for Gal1 in the mammalian circadian system, by characterizing the circadian activity behavior of the Gal1 null mutant (*Lgals1*^-/-^) [35].

## Methods

### Animals

*Lgals1*^-/- ^*mice *(C57BL/6J) were generated by F.P. at the Jacques Monod Institute (Paris), and were bred at the Animal facilities of the Faculty of Exact and Natural Sciences (University of Buenos Aires) and the Institute of Biology and Experimental Medicine (CONICET). Male mutants (n = 5) and male littermate wild-type mice (n = 4) were kept and manipulated according to the NIH Guide for the Care and Use of Laboratory Animals. The experimental protocol was approved by our institutional research committee. At 90 days of age, mice were placed in individual cages provided with a 17-cm running wheel, under a 12:12 light-dark schedule (LD), with water and food *ad libitum*. Mice were kept in constant darkness (DD) in order to characterize the endogenous circadian period.

### Wheel-running locomotor activity recording

Wheel-running activity was constantly monitored by a digital system (Archron, Argentina) which recorded the number of wheel revolutions every 5 minutes. Activity phase onset was taken as circadian time 12 (CT12). Light pulses (> 300 lux) were administered at CT 15 for the analysis of phase delays.

For reentrainment experiments, animals were initially maintained under a 12:12-h light-dark cycle (LD) for at least 10 days. Then, mice were subjected to an abrupt 6-h delay or advance in the phase of the LD cycle by shortening (for advances) or lengthening (for delays) the night previous to the shift. Time for reentrainment to the new LD cycle was defined as the time it took for each animal to adjust its activity onset with the new cycle according to the phase angle of the onset on the original LD scheme.

### Data analysis

All data presented is expressed as mean ± SEM. Activity data was analyzed using the tools included in El Temps software (Antoni Díez Noguera, University of Barcelona). Free-running activity periods were determined by Chi-square periodograms. To analyze alpha (the time devoted to nocturnal locomotion) and phase angle (the difference between activity onset and the time of lights off), mean waveforms were obtained from the activity recordings in the free-running interval and the "activity" period (alpha) was defined as the area of the curve on top of the median of activity. The area under the curve was calculated to analyze amplitude and activity concentration; amplitude was defined as the percent of total activity represented in the activity period, and concentration of activity was calculated as the ratio of this percent to alpha. In order to achieve a correct analysis of activity patterns, each individual waveform was built from relative activity data, obtained by determining the ratio of absolute activity counts to average counts in the analyzed period for each animal; this is later referred to as "Relative activity". Activity onset on each individual day was defined as the first 5-min bin that contained at least 80 wheel revolutions, followed by another bin of at least 80 wheel revolutions within 40 min. Phase shifts were calculated by measuring the phase difference between eye-fitted regression lines through 4-6 consecutive activity onset times immediately prior to the light pulse, and at least 10 consecutive activity onset times after the light pulse (excluding the 5 cycles immediately after the pulse) [36].

## Results

In classical behavioral tests, both wild-type and *Lgals1*^-/- ^mice exhibited normal entrainment to LD cycles, with a small but significant difference in phase angle: WT animals tended to start their locomotor activity before lights off while mutants presented their activity onset after ZT 12 (p < 0.05, Student's t test, Table 1). Upon transfer to DD conditions, the endogenous period of wheel-running activity was determined. Significant differences were found between circadian period for both groups; for WT mice, mean period was 23.57 ± 0.10 h, while for *Lgals1*^-/- ^mice was 24.02 ± 0.03 h (p = 0.002, Student's *t *test).

**Table 1:** Wheel-running activity recordings. Both *alpha *and phase shifts indicated are calculated relative to endogenous free-running period.

	Group		*P*
	
	Wild-type	Mutants	
Alpha in LD (h)	11.35 ± 0.62	12.12 ± 0.21	ns

Phase angle in LD (min)	-49 ± 23.15	9.4 ± 2.92	0.04

Free-running period (h)			
Before light pulse	23.57 ± 0.11	23.93 ± 0.03^†^	0.01
After light pulse	23.57 ± 0.10	24.02 ± 0.03^†^	0.002

Alpha in DD (circadian h)	12.35 ± 0.38	14.88 ± 0.88	0.04

Amplitude in DD (%)	97.3 ± 0.9	97.4 ± 0.5	ns

Amplitude/Alpha (%/h)	7.90 ± 0.27	6.62 ± 0.35	0.03

Phase delays in response to LP at CT15 (circadian h)	1.90 ± 0.34	2.92 ± 0.26	0.04

Reentrainment to 6 h advance of LD cycle (days)	8.7 ± 0.3	8.2 ± 0.4	ns

Reentrainment to 6 h delay (days)	2.0 ± 0.6	2.4 ± 0.4	ns

Phase angle in LD refers to the difference between the time of lights off and activity onset (negative values indicate animals start running before lights off). All data are expressed as Mean ± SEM. *P *values correspond to Student's *t *test. ^† ^Difference between these two results is not significant according to paired t test analysis.

**Figure 1:**
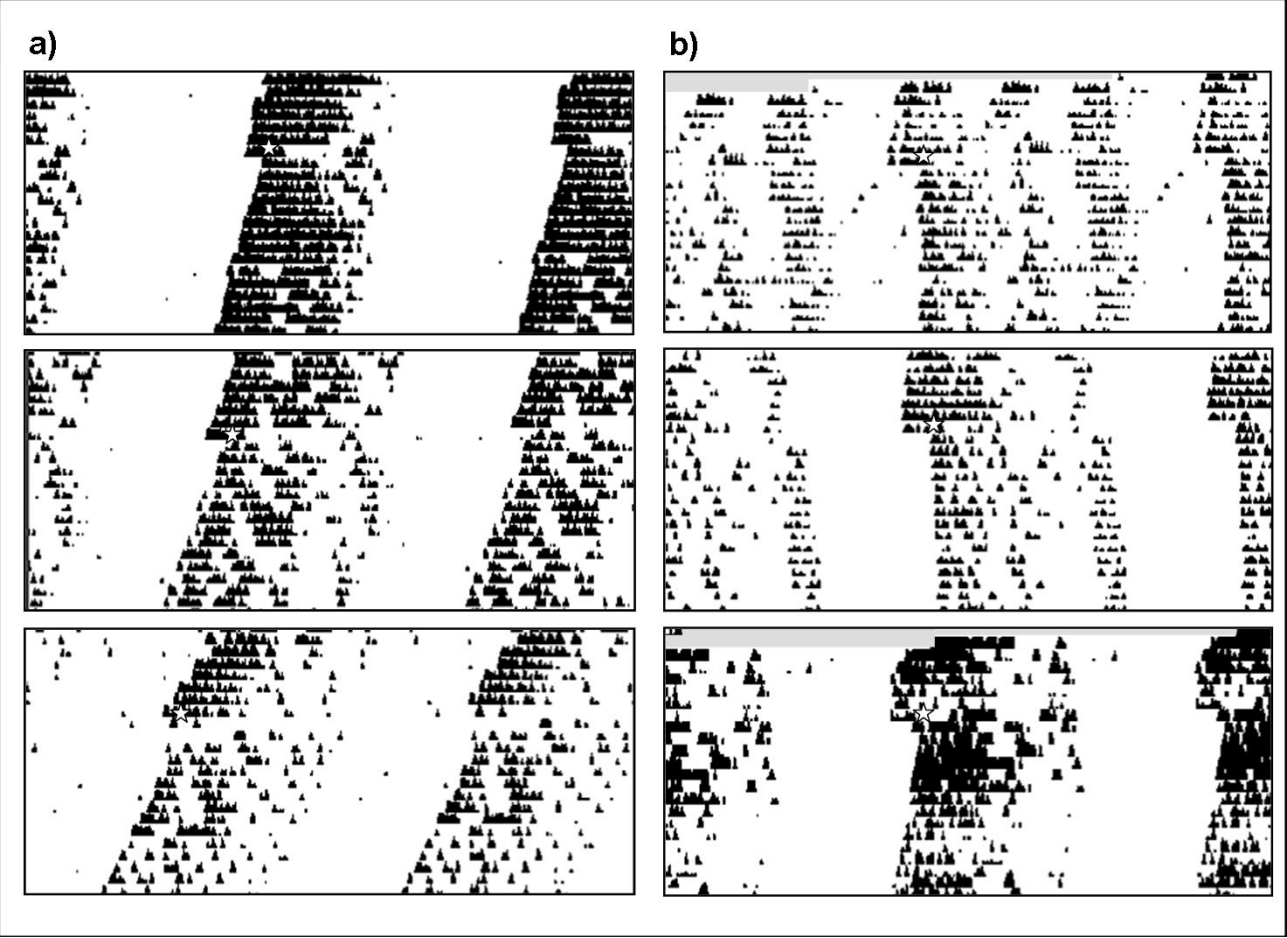
**Representative actograms of a) WT and b) *Lgals1*^-/-  ^type mice**. Stars indicate day and time of light pulses at CT15. Clear differences in free-running period and *alpha *can be appreciated (see Table 1). Moreover, after light pulse at CT 15 (i.e., 3 hours after activity onset under DD), a larger phase delay can be seen in *Lgals1*^-/- ^mice (see Table 1 for details).

**Figure 2:**
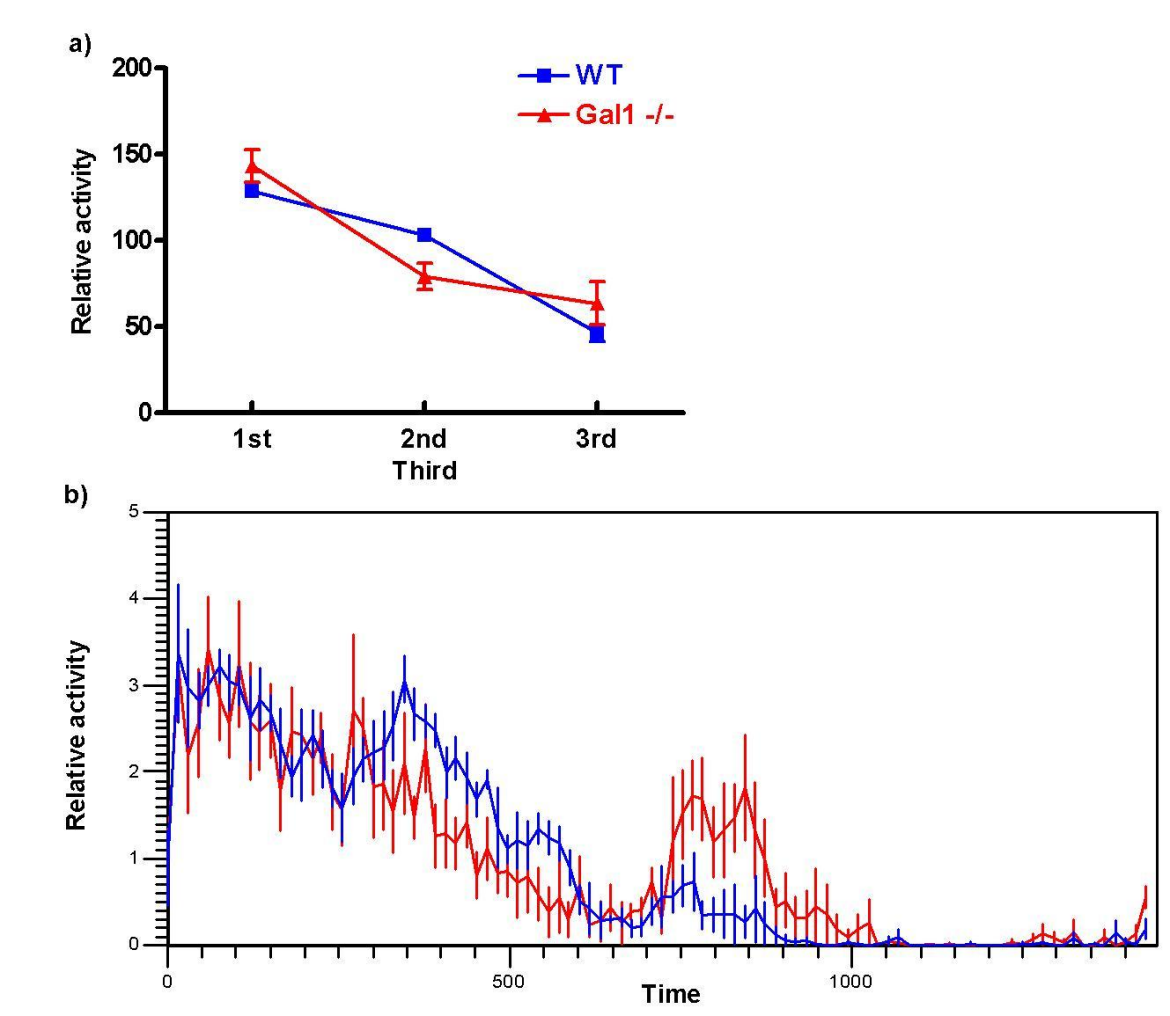
**a) Average amount of relative activity (see *Methods *for details) during each third of alpha, as determined from individual relative activity waveforms under free running conditions**. b) Average waveforms for each group, adjusted to *tau *period. Time point 0 represents CT12. All error bars represent mean ± SEM.

The length of the activity interval (i.e., *alpha*) was found to be significantly different between mutants and WT mice. Alpha for mutants was 14.88 ± 0.88 circadian hours while it was 12.35 ± 0.38 circadian hours for WT mice (p < 0.05, Student's *t *test). Amplitude was found to be similar in both groups, as activity intervals represented 97% of total activity. In consequence, the amount of activity as related to the total time spent in the activity interval was significantly higher in WT animals as compared to mutants. All data is summarized in Table 1, and representative actograms of both groups are shown in Fig. 1. A tendency towards a bimodal pattern in locomotor activity can be seen in mutant mice, although a three-block analysis of *alpha *revealed no significant differences between groups (Fig. 2).

Phase delays in response to light pulses at CT15 were significantly larger for mutant mice (2.92 ± 0.26 circadian h) than for WT animals (1.90 ± 0.34 circadian h) (p < 0.05, Student's t test). Data is summarized in Table 1 and representative actograms are shown in Fig. 1.

No significant differences were found between the groups for reentrainment to a phase delay or phase advance in the LD cycle. After a 6-h advance of the LD cycle, WT animals took 8.7 ± 0.3 days and mutants needed 8.2 ± 0.4 days for reentrainment. After 6-h delays the corresponding figures were 2.0 ± 0.6 and 2.4 ± 0.4 days, respectively. Low-amplitude phase advances were found for both WT and mutant mice when subjected to light stimulation at CT21, without any significant difference between them (data not shown).

## Discussion

According to our results, a deficit in Gal1 expression induces subtle, albeit significant, changes in the circadian behavior of mice. Mutant animals exhibited a longer circadian period and, as expected after this result, a larger phase delay response to light pulses (according to the phase response curve of mice; larger phase delays would allow animals with a longer endogenous period to entrain to a 24-h LD cycle). However, the similarities between reentrainment rate between mutant and WT animals suggest that Gal1 might act on a non-parametric photic mechanism (i.e., the response to light pulses) rather than a parametric effect, such as a complete photoperiod synchronization.

In addition, we have preliminary evidence for Gal-1 expression in the suprachiasmatic region of control mice, which was absent in knockout animals (see additional File 1: Immunohistochemical analysis of Gal-1 in the suprachiasmatic area). Indeed, the expression of Gal1 in the suprachiasmatic nuclei (SCN) region of the CNS would suggest that this factor might be implicated in both the generation and the entrainment of circadian rhythms. It is tempting to speculate that the lack of Gal1 might affect the normal development of SCN neurons, as has been found for other CNS areas such as the olfactory bulb [37-39]. It is possible that small structural differences along neural developmental may elicit changes in the adult circadian behavior and responses to light.

Moreover, Gal1 expression in glial cells [28,29,40,41] provides an additional substrate for immune-related effects on circadian responses, since glial cells have been implicated in the generation and modulation of circadian rhythms [16,42]. In particular, astrocytic-derived Gal1 has also been reported to modulate glutamate neurotoxicity by altering the expression of NMDA receptors [31], which are key actors in the signal transduction pathway for circadian responses to light [43]. Moreover, changes in these receptors, present in the retinorecipient portion of the SCN and responsive to photic stimulation, might explain at least in part the variation in light-induced phase shifts in the mutants.

## Conclusions

The differences reported for circadian period and light-induced phase delays in Gal1 mutants should also be taken into account when employing this model in immune or behavioral research. Further research of daily or circadian fluctuations in specific immune variables should be performed in order to establish the possible implications of Gal1 in the mammalian circadian system. To illustrate this concept, Gal1-mediated tolerogenic mechanisms, including promotion of T cell apoptosis, modulation of Th2 cytokine balance or the control of dendritic cell physiology may be selectively influenced by the circadian system. This implies caution in the monitoring of these immunoregulatory effects which may substantially fluctuate during the light-dark cycle. In conclusion, we provide the first evidence of alterations in the behavior of mice devoid of Gal1, which prompts further investigation of the relevance of this endogenous protein in physiopathological processes.

## Competing interests

The authors declare that they have no competing interests.

## Authors' contributions

LPC performed the behavioral experiments and analysis; DOC performed the immunohistochemistry; FP generated and provided the mutant mice; LPC, GAR and DAG designed the experiments, analyzed the results and wrote the manuscript. All authors approved the final version of the manuscript.

## Supplementary Material

Additional file 1 **Immunohistochemical analysis of Galectin-1 in the suprachiasmatic area**. Brains from 3 WT or 2 Gal-1 deficient (*Lgals1*^-/-^) mice housed under 12:12 LD were perfused at zeitgeber time 6 with paraformaldehyde 4% and stored in PBS/sucrose until use. Sections (40-50 μm) were cut in vibratome and incubated in PBS 10% normal goat serum for 1 h at room temperature. Samples were then incubated with anti-Gal1 rabbit polyclonal IgG (1:200 dilution) used as described [22,44] or control pre-immune rabbit IgG (same dilution), in PBS containing 2% goat serum overnight at 4°C. Subsequently, samples were rinsed in PBS for 30 min and then incubated with FITC-conjugated goat anti-rabbit IgG (BD Bioscience 1:200) for 2 h at room temperature. Following extensive washing, samples were mounted in anti-fading solution on glass slides and analyzed on a Nikon E800 scanning laser confocal microscope. As a control, primary antibody was omitted in some sections and processed as described above. Cellular nuclei were stained with propidium iodine. To determine whether Gal1 plays a role in the regulation of mice circadian behavior, we evaluated expression of this glycan-binding protein in the suprachiasmatic region of the brain. In the pictures, propidium iodine-stained nuclei are shown in red and Gal-1 expression is stained in green. A widespread and specific immunoreactivity of Gal-1 was observed (A), in contrast to what has been reported for *Lgals1*^-/- ^mice [35]. In addition, strong gal-1 expression was found in the olfactory bulb of wild-type mice (data not shown). (B) As a control, *Lgals1*^-/- ^showed no expression of galectin-1. Click here for file
