# Supplementary figures and images for: "Time sweet time": circadian characterization of galectin-1 null mice
Source: J Circadian Rhythms. 2010 Apr 19;8:4. doi: 10.1186/1740-3391-8-4 (PMC2876058; doi:10.1186/1740-3391-8-4)

A)

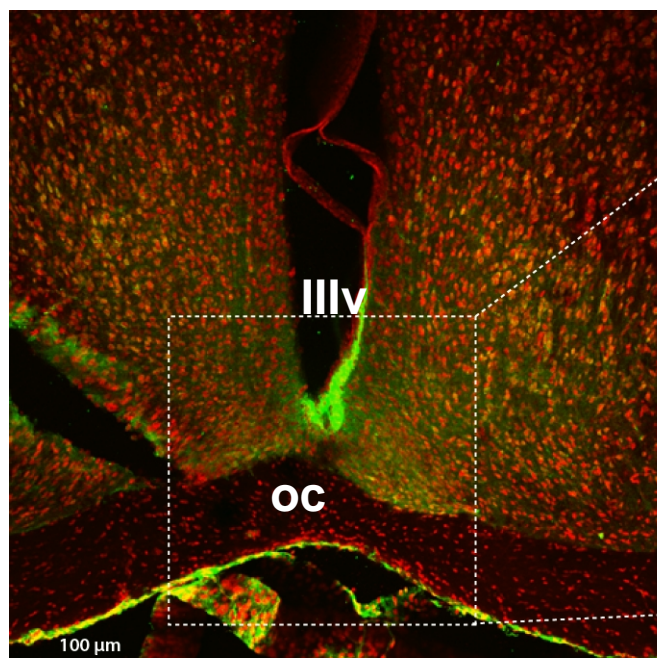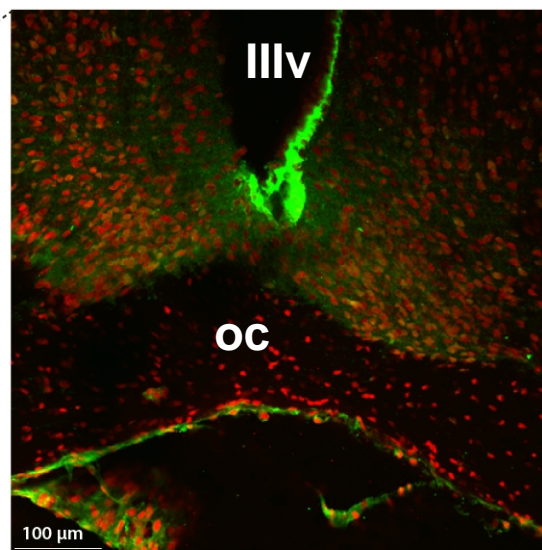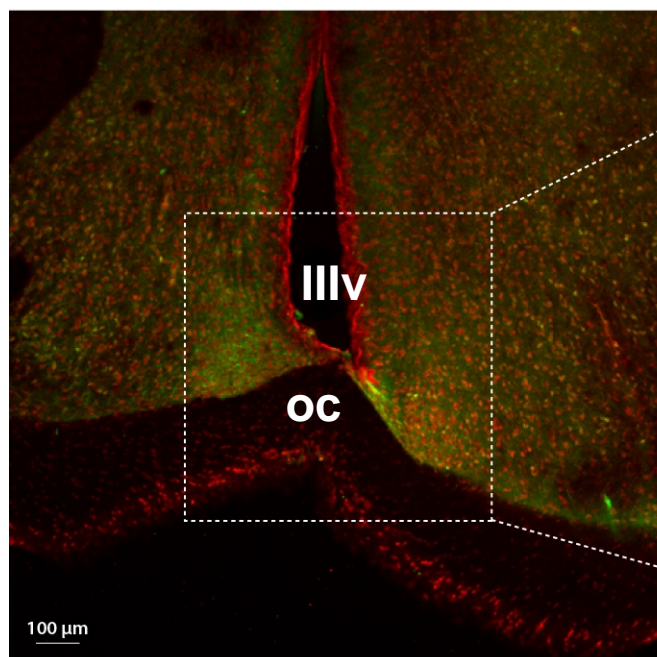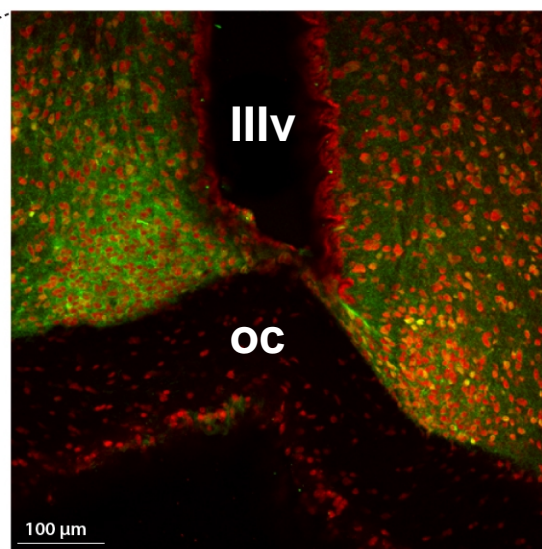

B)

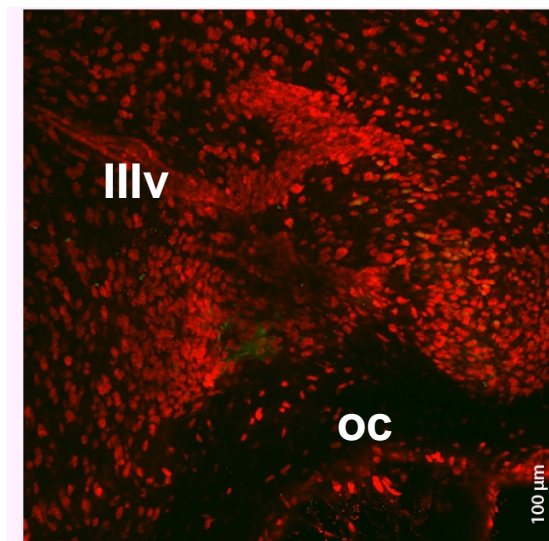

Supplement: Additional file 1 — Immunohistochemical analysis of Galectin-1 in the suprachiasmatic area. Brains from 3 WT or 2 Gal-1 deficient (Lgals1-/-) mice housed under 12:12 LD were perfused at zeitgeber time 6 with paraformaldehyde 4% and stored in PBS/sucrose until use. Sections (40-50 μm) were cut in vibratome and incubated in PBS 10% normal goat serum for 1 h at room temperature. Samples were then incubated with anti-Gal1 rabbit polyclonal IgG (1:200 dilution) used as described [22,44] or control pre-immune rabbit IgG (same dilution), in PBS containing 2% goat serum overnight at 4°C. Subsequently, samples were rinsed in PBS for 30 min and then incubated with FITC-conjugated goat anti-rabbit IgG (BD Bioscience 1:200) for 2 h at room temperature. Following extensive washing, samples were mounted in anti-fading solution on glass slides and analyzed on a Nikon E800 scanning laser confocal microscope. As a control, primary antibody was omitted in some sections and processed as described above. Cellular nuclei were stained with propidium iodine. To determine whether Gal1 plays a role in the regulation of mice circadian behavior, we evaluated expression of this glycan-binding protein in the suprachiasmatic region of the brain. In the pictures, propidium iodine-stained nuclei are shown in red and Gal-1 expression is stained in green. A widespread and specific immunoreactivity of Gal-1 was observed (A), in contrast to what has been reported for Lgals1-/- mice [35]. In addition, strong gal-1 expression was found in the olfactory bulb of wild-type mice (data not shown). (B) As a control, Lgals1-/- showed no expression of galectin-1. [file 1740-3391-8-4-S1.PDF]
